# The validity and reliability of the Patient Health Questionnaire-9 for screening depression in primary health care patients in Botswana

**DOI:** 10.1186/s12888-020-02719-5

**Published:** 2020-06-12

**Authors:** Keneilwe Molebatsi, Keneilwe Motlhatlhedi, Grace Nduku Wambua

**Affiliations:** 1 Department of Psychiatry, Faculty of Medicine, University of Botswana, Private Bag, 00713 Gaborone, Botswana; 2 Department of Family Medicine and Public Health, Faculty of Medicine, University of Botswana, Gaborone, Botswana; 3 Department of Psychiatry, College of Health Sciences, University of KwaZulu Natal, Durban, South Africa

**Keywords:** Patient Health Questionnaire-9, Primary health care, Botswana, Depression, Validity, Reliability

## Abstract

**Background:**

The lack of locally validated screening instruments contributes to poor detection of depression in primary care. The Patient Health Questionnaire-9 (PHQ-9) is a brief and freely available screening tool which was developed for primary care settings; however, its accuracy may be affected by the population in which it is administered. This study aimed to determine the validity and reliability of PHQ-9 for screening depression in a primary care population in Botswana.

**Methods:**

Data was collected from a conveniently selected sample of 257 adult primary care attendants. The Mini International Neuropsychiatric Interview (MINI) depression module was used as a gold standard to assess criterion validity.

**Results:**

Sensitivity and specificity of the PHQ-9 for screening for major depression were 72.4 and 76.3 respectively at a cut off score of nine or more. The area under the ROC curve was 0.808. The PHQ-9 demonstrated good internal consistency with a Cronbach alpha of 0.799. Criterion validity was demonstrated by significant correlation (*r* = 0.528, *p* < 0.001) between PHQ-9 and the MINI. Significant negative correlation between PHQ-9 scores and all four domains of the WHO quality of life questionnaire- brief version scores demonstrated good convergent validity.

**Conclusions:**

The PHQ-9 is a reliable and valid instrument to screen for depression in primary care facilities in Botswana. Primary care clinicians in Botswana may use the PHQ-9 to screen for depression with a cut –off score of nine. Further studies should focus on integrating routine depression screening in primary care.

## Background

Depression is an important public health problem. It is a disabling chronic mental health problem commonly encountered in primary care. According to the World Health Organization (WHO) estimates, unipolar depressive disorders will become the leading cause of the global burden of disease by 2030 (World Federation for Mental Health (WFMH), 2012).

The prevalence of depression is markedly higher in people with other medical conditions such as diabetes mellitus and cardiovascular diseases, for example: 17 to 28% of patients with cardiovascular conditions and diabetes mellitus have been found to have depression [1, 2]. The co-morbidity of chronic conditions and depressions is associated with worse outcomes [3, 4]. Because most people with chronic diseases are diagnosed and managed in primary care, detecting depression at this early stage may improve outcomes.

WHO mental health GAP Intervention Guide has shown that depression can be reliably diagnosed and treated in primary care [5]. Despite treatment for depression in primary care being feasible, affordable and cost-effective [6]; the condition is often underdiagnosed and undertreated in primary care settings, especially in low to middle income countries (LMIC) [7]. Factors such as: inadequate numbers of specialised mental health workers, stigma associated with mental illness, and lack of locally validated screening instruments contribute to poor rates of diagnosis and treatment of depression in primary care.

Screening tools, if used appropriately may help health care workers to accurately identify patients with depressive disorders and initiate appropriate management. There are several screening and diagnostic tools available for depression [8, 9], however, not all of the instruments are suitable for use in primary care settings. The use of some depression screening and or diagnostic tools is limited by cost, time needed to administer the tools and in the case of self-administered tools, the patient’s ability to accurately complete the tool.

The Patient Health Questionnaire-9 (PHQ9) was specifically developed for use in primary care settings [10], is available at no cost and has been validated in several settings to screen for depression [9–12]. As with many diagnostic tools the accuracy of the PHQ-9 may be affected by the population in which it is administered [13, 14]. Although a study of the PHQ-9 amongst 4 different ethnic groups found that the interpretation of the PHQ-9 did not differ amongst the ethnic groups, all four groups were in America and could have similar understanding of depression based on a shared environment [15]. It is therefore important to determine the optimal cut-off point when screening for depression using the PHQ-9 in a specific population.

The aim of the present study was to evaluate the validity and reliability of an interviewer administered PHQ-9 for screening depression among primary care patients in Botswana. The MINI major depressive episode module was used as a reference standard. This is the first study to investigate the reliability and validity of a depression screening tool in Botswana.

## Methods

### Study design and site description

The study utilised a cross sectional design. The study was conducted at two primary care facilities in Gaborone, the capital and largest city in Botswana (population ~ 200,000) [16]. The two facilities were purposively selected to make the sample heterogeneous. One facility is located in a low-income area and the other in a predominantly middle to high income area. The two facilities also have a psychiatric nurse on site, who were crucial in providing further management to study participants who were found to have depression.

### Study population

The study targeted adult patients attending the selected clinics during the months of May and June 2019. Convenience sampling was used to select potential participants, selecting patients in the order they came to the clinic. To be included in the study, patients had to be 18 years or older and be able to comprehend and complete study components in Setswana or English. Patients with moderate to severe intellectual disability, experiencing acute psychotic symptoms and having any unstable medical condition were not included in the study.

### Measures

#### Researcher designed socio demographic clinical questionnaire

The researchers designed an instrument to capture social and demographic data such as gender and income. The instrument also screened for history of chronic medical conditions including hypertension, cancer, Human Immunodeficiency Virus (HIV), and family history of any mental illness.

#### Patient Health Questionnaire (PHQ-9)

For this study we used the English version of the PHQ-9 by Kroenke et al. to screen for depression [10]. The PHQ-9 is a 9-item instrument used to screen for depression. Respondents indicate frequency of depression symptoms in the preceding 2 weeks on a 4-point scale, ranging from 0 (never) to 3 (nearly every day), for a total score ranging from 0 to 27, with higher scores indicating increased likelihood of a major depressive disorder. Total scores are interpreted as follows: Minimal depression (0–4), Mild depression (5–9), Moderate depression (10–14) and Severe depression (20–27) [10]. In the study by Kroenke et al. (2001); total scores of 10 or more on the PHQ-9 predicted a depressive disorder at a sensitivity of 88% and a specificity of 88%. The PHQ-9 demonstrated an excellent internal reliability with a Cronbach’s α of 0.89 [10]. The PHQ-9 has been used previously in Botswana [17].

#### Mini International Neuropsychiatric Interview (MINI)

The Mini International Neuropsychiatric Interview (MINI) is a short diagnostic structured interview developed in Europe and the United States to explore 17 psychiatric disorders [18, 19]. It is fully structured to allow administration in about 15 to 20 min even by non-specialized interviewers [20, 21]. The MINI-plus demonstrates good sensitivity, specificity, validity and reliability in the assessment of psychiatric disorders [18, 20, 21]. The major depressive episode module was used in this study as a gold standard.

#### World Health Organization Quality of Life scale Brief Version (WHOQOL-BREF)

The instrument is used to assess cross cultural quality of life across four domains: Physical health, psychological health, social relationships and environmental health. It assesses the respondent’s perceptions in the context of their standards, concerns and culture [22]. Respondents are requested to endorse perceived quality of life on a 5-point likert scale ranging from 1 (not at all) to 5 (completely) with higher scores indicating a higher quality of life [23]. The WHOQOL-BREF has demonstrated good to excellent psychometric properties and is reliable with a Cronbach α of more than 0.7 for all domains [23].

### Data collection procedures

Research assistants were trained by the authors to administer the English and Setswana versions of socio-demographic questionnaire, the PHQ-9 and the WHOQOL-BREF and the MINI major depressive episode module.

Participants were recruited conveniently by selecting consecutive patients from the consultation waiting room. Those interested to participate in the study were directed to a research assistant in a private room. A written informed consent was obtained before commencing with data collection. Each participant was sequentially interviewed by two research assistants in either Setswana or English according to the participant’s preference. The first research assistant administered the socio-demographic questionnaire and the PHQ-9. Upon completion of the first interview each participant was then directed to a different room where the second research assistant (a Bachelor of Psychology graduate) who was blinded to the results of the PHQ-9 screening, then administered the MINI major depressive episode module and the WHOQOL-BREF.

Participants who were found to be suicidal or who screened positive for depression were referred to a psychiatric nurse stationed at the facility for further management.

### Data analysis

Items means, standard deviations, frequencies and percentages were calculated for the socio-demographic variables. Independent samples t-tests was carried out to compare the mean PHQ-9 scores in the groups of depressed and not depressed patients according to MINI-Plus diagnosis.

#### Reliability

In order to investigate the reproducibility and consistency of PHQ-9, reliability coefficients as measured by Cronbach’s alpha were calculated.

#### Concurrent and convergent validity

Using spearman’s correlation analysis, the relationship between scores of PHQ-9 and MINI-plus diagnosis was investigated to determine the magnitude of the relationship between the two measures. Convergent and concurrent validity require that PHQ-9 should correlate with MINI-Plus diagnosis whilst it inversely correlates with quality of life as determined by the WHOQoL-BREF.

#### Factor structure

Before performing factor analysis, the correlation matrix was inspected to check for the strength of correlation and then factorability was tested using explanatory factor analysis using Kaiser-Meyer-Olkin (KMO) measure of sampling adequacy and Bartlett’s test of sphericity. We calculated KMO = 0.838 and Bartlett’s test of sphericity (469.303, df = 36, *p* < 0.001) making factor analysis an appropriate method.

#### Sensitivity and specificity

For each PHQ-9 cutoff point, sensitivity or true positive rate (proportion of individuals with MDE according to MINI criteria that correctly identified by PHQ-9), specificity or true negative rate (proportion of individuals without MDE according to the gold standard correctly identified as such by PHQ-9), Positive predictive value +PPV (proportion of true positives among all positives identified by the PHQ-9) and Negative predictive value -NPV (proportion of true negatives among all those who will score negative by PHQ-9 was calculated. Positive likelihood ratio + LR (probability of an individual without the condition having a positive test) and Negative likelihood ratio –LR (probability of an individual without the condition having a negative test) 95% confidence intervals for each of these parameters was reported.

Youden’s index was used as a criterion for choosing the “optimal” threshold value for the PHQ-9 test, the threshold value for which the value of [sensitivity + specificity − 1] is maximized.

Criterion validity was assessed by receiver operating characteristic (ROC) curve. The PHQ-9 point showing simultaneously the highest sensitivity and specificity was evaluated using the ROC curve. PHQ-9’s accuracy was estimated by the area under the ROC curve. All analyses were performed using Stata® version 14.0 software.

## Results

### Study sample characteristics

Majority of the participants in our study were female at 66.9% (*n* = 172) and the mean age of our participants was 34.3 years. The most common chronic diagnosis was HIV at 21.6% (*n* = 60) and alcohol was the most commonly used substance. Only 2.4% of the study participants had previously been diagnosed with a mental illness. Other characteristics are as depicted in Table 1.

**Table 1:** Participants characteristics

Variable	Category	Frequency (***N*** = 257)	Percentage (%)
Gender	Female	172	66.9
	Male	85	33.1
Age	Mean; Median; Range	34.3; 32.0; 18–79	
Age	18–24 Years	70	27.2
	25 and Above	187	72.8
Marital status	Single	63	24.6
	In a relationship	87	34.0
	Married	29	11.3
	Widowed	8	3.1
	Divorced	10	3.9
	Cohabiting	59	23.0
	*Missing*	1	
Employment status	Unemployed	60	23.3
	Self Employed	35	13.6
	Part Time	9	3.5
	Casual labourer	9	3.5
	Full time Employment	125	48.6
	Student	19	7.4
Religion^a^	Islam	4	1.5
	Christianity	226	87.3
	African Tradition religion	13	5.0
	Other	15	5.8
	None	1	0.4
	*Missing*	2	
Education Level	Never went to school	6	2.3
	Primary	29	11.3
	Junior Secondary	85	33.1
	Senior Secondary	73	28.4
	Tertiary	64	24.9
Diagnosis^a^	HIV	60	21.6
	Hypertension	44	15.8
	Stroke/CVA	6	2.2
	Diabetes	5	1.8
	Other	21	7.6
	None	142	51.1
	*Missing*	4	
Diagnosis	None	142	56.1
	One	89	35.2
	Multiple/Comorbid	22	8.7
	Missing	4	
Income^a^	Below P600	45	17.6
	600–2399	106	41.4
	2400–4199	44	17.2
	4200–6000	23	9.0
	Above 6000	19	7.4
	Not Disclosed	19	7.4
	*Missing*	1	
Substance Use	Alcohol	110	37.4
	Dagaa	14	4.8
	Tobacco	31	10.5
	other-Name	2	0.7
	No	137	46.6
	*Missing*	2	
Substance Use	None	137	53.7
	One	88	34.5
	Multiple	30	11.8
	*Missing*	2	
Relative with mental Illness	Yes	59	23.2
	No	179	70.5
	Don’t Know	16	6.3
	*Missing*	3	
Relative with mental illness	No	195	76.8
	Yes	59	23.2
	*Missing*	3	
History of mental illness	Yes	6	2.4
	No	249	97.6
	*Missing*	2	
	*Missing*		2

^a^The numbers do not add up to 257 because the participants answered more than one option

### Prevalence of depression

**Fig. 1:**
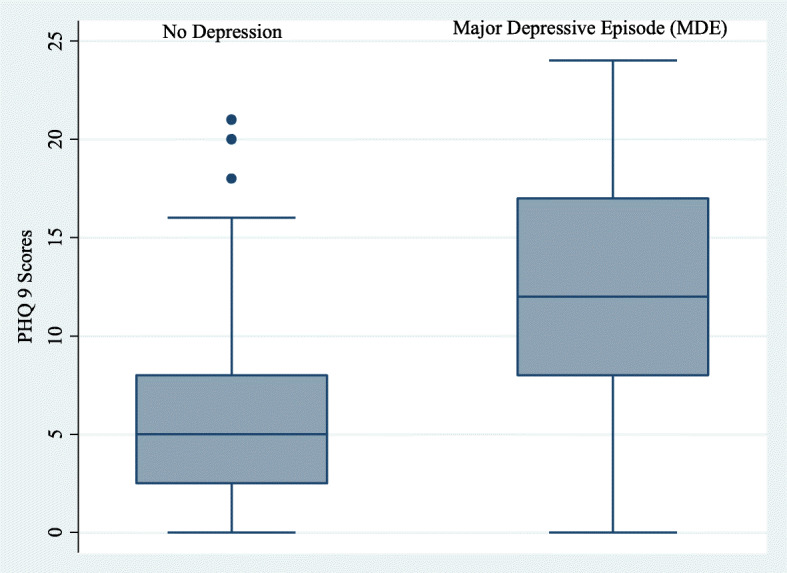
Box diagram and distribution scores of the PHQ-9 scores for participants with depression and those without depression

A total of 105 participants fulfilled the DSM-IV criteria for a major depressive episode, using the gold standard MINI depression module corresponding to a prevalence of 40.9% (95% C. I 35.0–47.1). The mean depression scores in the PHQ-9 were significantly higher among the depressed as compared to the non-depressed as shown in Fig. 1.

### Reliability of PHQ-9

Table 2 presents the results of reliability. The PHQ-9 demonstrated good internal reliability with a Cronbach’s alpha of 0.799.

**Table 2:** Reliability of PHQ-9

PHQ-9 Items	PHQ-9 Questions	Scale Mean if Item Deleted	Corrected Item-Total Correlation	Cronbach’s Alpha if Item Deleted	Items Mean ± SD
PHQ-1	Little interest or fun in doing things	7.80	0.540	0.773	0.90 ± 1.08
PHQ-2	Feeling down, depressed or hopeless	7.34	0.602	0.764	1.36 ± 1.17
PHQ-3	Trouble falling asleep, staying asleep or sleeping too much	7.44	0.484	0.781	1.26 ± 1.23
PHQ-4	Feeling tired or having little energy	7.62	0.456	0.784	1.08 ± 1.15
PHQ-5	Poor appetite or overeating	7.66	0.494	0.779	1.04 ± 1.19
PHQ-6	Feeling bad about yourself, or that you’re a failure or have let yourself or your family down	7.57	0.456	0.784	1.13 ± 1.15
PHQ-7	Trouble concentrating on things such as reading the newspaper or watching television	7.68	0.522	0.775	1.02 ± 1.11
PHQ-8	Moving or speaking so slowly that other people could have noticed. Or the opposite	8.15	0.404	0.790	0.55 ± 0.97
PHQ-9	Thoughts that you would be better off dead or hurting yourself in some way	8.33	0.466	0.784	0.37 ± 0.82
**Overall**	**Cronbach’s Alpha**	**0.799**			8.65 ± 6.1

### Sensitivity and specificity

**Fig. 2:**
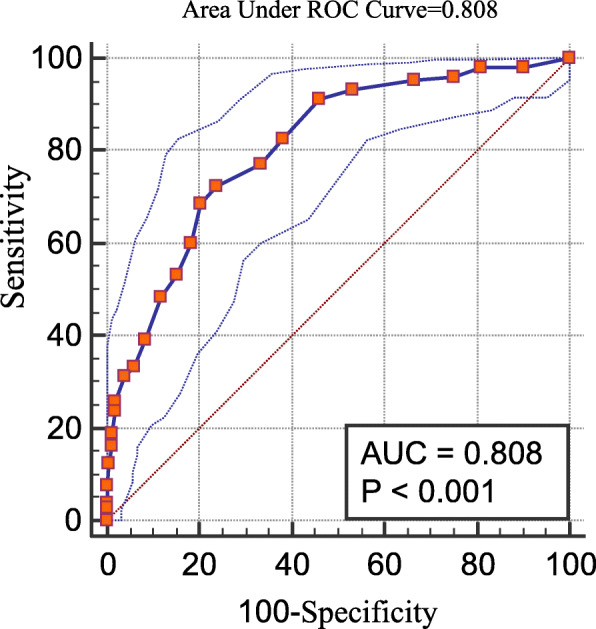
Receiver operator characteristics curve for the performance of Patient Health Questionnaire Scale (PHQ-9) compared to MINI

Table 3 shows sensitivity, specificity, positive and negative likelihood ratio, positive predictive value and negative predictive value for each of the PHQ-9 cutoff points compared to the gold standard interview (MINI). As expected, sensitivity decreased progressively as the cutoff points increased, with a marked decrease between the ≥10 and ≥ 11 cutoff points (from 68.6 to 60.0%) while specificity between these two cutoff points increased from 79.6 to 81.6%. The standard cut off score of 10 or higher yielded a sensitivity of 68.6 and a specificity of 79.6. Both Youden’s index and the cutoff point of maximum sensitivity and specificity according to the Receiver Operating Curve (ROC) (Fig. 2) indicate the ≥9 cutoff point as most suitable for identifying individuals at increased risk of having depression in this population. At total of 112 individuals (43.6%; 95% C. I 37.4–49.4) scored ≥9 in the PHQ-9 scale. Sensitivity at this point was 72.4% (95% C. I 62.8–80.7) and specificity of 76.3% (95%CI 68.7–82.5).

**Table 3:** Sensitivity, specificity, +LR, −LR, PPV, NPV, accuracy for different PHQ-9 cut-off points compared to the gold standard MINI and Youden’s index

Cut-off Points	N(%)	Sensitivity (95% C.I)	Specificity (95% C.I)	+LR (95% C.I)	-LR (95% C.I.)	PPV (95% C.I)	NPV (95% C.I)	Youden’s Index^**a**^
≥5	179 (69.6%)	93.3 (86.7–97.3)	46.7 (38.6–55.0)	1.8 (1.5–2.0)	0.14 (0.07–0.3)	54.7 (50.8–58.6)	91 (82.9–95.5)	0.4004
≥6	166 (64.6%)	91.4 (84.4–96.0)	54.0 (45.7–62.1)	2.0 (1.7–2.4)	0.16 (0.08–0.3)	57.8 (53.3–62.2)	90.1 (82.7–94.5)	0.4538
≥7	145 (56.4%)	82.9 (74.3–89.5)	61.8 (53.6–69.6)	2.2 (1.7–2.7)	0.28 (0.2–0.4)	60 (54.6–65.2)	83.9 (77.1–89.0)	0.4470
≥8	132 (51.4%)	77.1 (67.9–84.8)	66.5 (58.3–73.9)	2.3 (1.8–2.9)	0.34 (0.2–0.5)	61.4 (55.4–67.0)	80.8 (74.4–85.9)	0.4359
**≥9**	**112 (43.6%)**	**72.4 (62.8–80.7)**	**76.3 (68.7–82.8)**	**3.1 (2.2–4.2)**	**0.36 (0.3–0.5)**	**67.9 (60.8–74.2)**	**80 (74.3–84.7)**	**0.4870**
≥10	103 (40.1%)	68.6 (58.8–77.3)	79.6 (72.3–85.7)	3.4 (2.4–4.7)	0.39 (0.3–0.5)	69.9 (62.3–76.5)	78.6 (73.2–83.1)	0.4818
≥11	91 (35.4%)	60.0 (50.0–69.4)	81.6 (74.5–87.4)	3.3 (2.3–4.7)	0.49 (0.4–0.6)	69.2 (60.9–76.5)	74.7 (69.8–79.1)	0.4158
≥12	79 (30.7%)	53.3 (43.3–63.1)	84.9 (78.2–90.2)	3.5 (2.3–5.3)	0.55 (0.4–0.7)	70.9 (61.6–78.7)	72.5 (68.0–76.6)	0.3820
≥13	69 (26.8%)	48.6 (38.7–58.5)	88.2 (81.9–92.8)	4.1 (2.5–6.6)	0.58 (0.5–0.7)	73.9 (63.8–82.0)	71.3 (67.1–75.1)	0.3673
≥14	54 (21.0%)	39.1 (29.7–49.1)	91.5 (85.8–95.4)	4.6 (2.6–8.1)	0.67 (0.6–0.8)	75.9 (64.0–84.8)	68.5 (64.9–71.8)	0.3050

*LR +* Positive likelihood ratio, *LR*– Negative likelihood ratio, *PPV* Positive predictive value, *NPV* Negative predictive value; (^a^) Youden’s Index = [Sensitivity + Specificity-1]

The PHQ-9 demonstrated very good accuracy with an area under the ROC (AUC) of 0.808 (95% C. I 0.755–0.854) as depicted in Fig. 2.

### Concurrent and convergent validity

The PHQ-9 scores correlated with the results of MINI (*r* = 0.528, *p* < 0.001). As expected, higher PHQ-9 score correlated with lower quality of life in all domains of the WHOQoL-BREF. The correlation between PHQ-9 scores and WHOQoL-BREF scores on all the four domains were found to have a significant negative correlation in all the four domains of WHOQoL-BREF (Table 4).

**Table 4:** Correlations between PHQ-9 depression scores, quality of life domain scores and depression

Pearson’s Correlations	1	2	3	4	5	6
1. PHQ-9 Depression Scores	1					
2. Physical Quality of Life	**−0.330**^**a**^	1				
3. Psychological Quality of Life	**−0.468**^**a**^	0.348^a^	1			
4. Social Quality of Life	**−0.411**^**a**^	0.377^a^	0.414^a^	1		
5. Environmental Quality of Life	**−0.499**^**a**^	0.403^a^	0.483^a^	0.529^a^	1	
6. Major Depression Episode^b^	**0.525**^**a**^	−0.244^a^	−0.346^a^	−0.299^a^	−0.389^a^	1

^a^ Correlation is significant at the 0.01 level (2-tailed), ^b^ Spearman’s rho Correlation

### Exploratory factor analysis

**Fig. 3:**
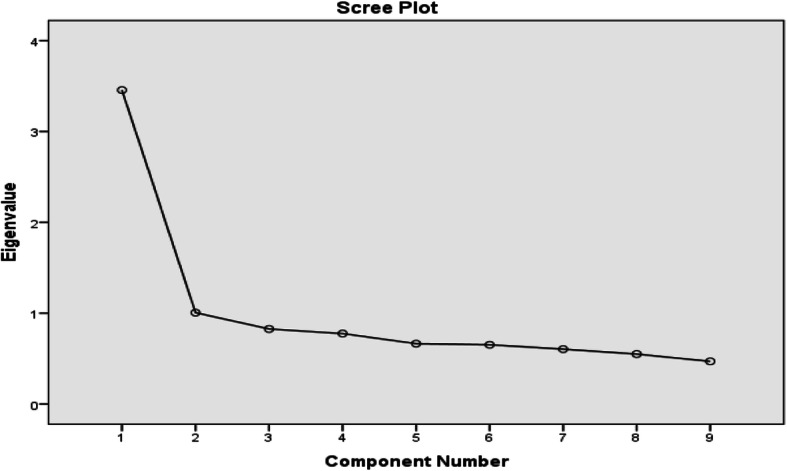
Scree-plot

The Kaiser-Meyer-Olkin (KMO = 0.838). and Bartlett test of sphericity (469.308, df = 36, *P* < 0.001) justified a dimension reducing procedure such as the factor analysis. The measure of sampling adequacy was > 0.80, so the items could be considered suitable for factor analyses. The scree plot (Fig. 3) revealed one dominant dimension with a big decrease between first and second eigenvalues and small decreases afterward (eigenvalues: 3.4, 1.0, 0.8, 0.7, 0.66, 0.65, 0.6, 0.5 and 0.469). Factor loadings ranged from 0.54 to 0.82. The percentage of total variance explained by the first factor was 38.4% and the second factor explained 11.2% of the variance.

**Fig. 4:**
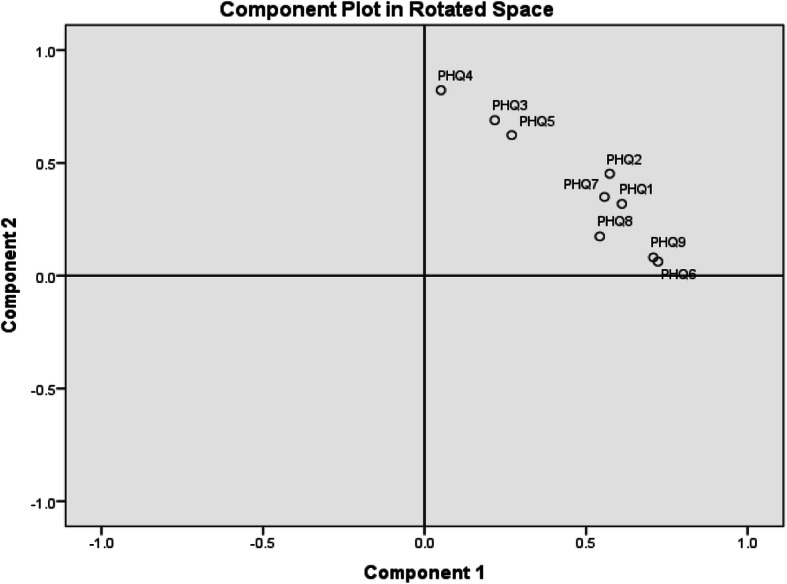
Component Plot in Rotated Space

These two factors explained 49.6% of the variance as shown in the scree plot graph (Fig. 3). Factor 1 included questions 1, 2, 6, 7, 8, and 9 and factor two 3, 4, and 5 (Table 5). These factors did not reflect the multidimensional structure PHQ-9. However, component rotated space (Fig. 4) revealed only one factor and to judge the strength of the measurement dimension, the following cut off points were used for variance explained by the measure: > 40% is considered a strong measurement dimension, > 30% is considered a moderate measurement dimension, and > 20% is considered a minimal dimension. Factor 1 explained approximately 40% of the variance which is supportive of unidimensionality.

**Table 5:** Exploratory factors loadings and explained variance after rotation for the PHQ-9 items

Factors		Rotation Sums of Squared Loadings				
		Factor Loadings	Eigen Values	% of Variance	Cumulative %	Cronbach’s Alpha
Factor 1	PHQ-1	0.611	3.456	38.394	38.394	0.749
	PHQ-2	0.573				
	PHQ-6	0.722				
	PHQ-7	0.557				
	PHQ-8	0.542				
	PHQ-9	0.708				
Factor 2	PHQ-3	0.689	1.006	11.173	49.567	0.615
	PHQ-4	0.823				
	PHQ-5	0.623

## Discussion

Depression is one of the leading causes of disability [24] and the second leading cause of years of life lived with disability [25]. However, depression still remains under-recognized in primary care settings [26]. Identifying depression in primary care patients can be a challenge, especially in busy clinics. A short and valid screening tool can assist in the identification of patients with depression in different primary care settings. This study aimed to estimate the reliability and validity of PHQ-9 for the identification of depression in patients attending primary health care facilities in Botswana.

Using the gold standard MINI depression module, 40.9% (95% C. I 35.0–47.1) of the study population met the criteria for current Major Depressive Episode. This was higher than the prevalence of 38% in an earlier study of HIV-infected patients in Botswana [27] and the prevalence of 30.3% found among primary care patients in Malawi [28], but lower than 56% in a South African Primary care population [29]. The variance in the studies could be explained by the different depression diagnostic tools used in the studies. Additionally, factors such as HIV status, socio-economic status and cultural influences on the understanding of depression may influence the results.

The internal consistency analysis of the PHQ-9 was assessed using the Cronbach’s alpha coefficient, and it was found to be 0.799. This indicates that the PHQ-9 has acceptable predictive performance. Our findings were comparable or slightly lower to that seen in validation studies in other parts of sub-Saharan Africa whose Cronbach alpha values were found to be 0.76 in South Africa [11], 0.83 in Malawi [30] and 0.85 in Ethiopia [31].

When the PHQ-9 was compared with the gold-standard diagnosis of the MINI, it performed well showing reasonable accuracy in identifying cases of depression. The area under the ROC curve was found to be 0.808, suggesting good diagnostic ability of the PHQ-9 score. Kroenke at al., (2001) used a cut off, of 10, with a sensitivity of 88% and specificity of 88%. We found that, using the PHQ-9, depression was best identified by a cut off nine. At this cut off, the PHQ-9 had moderately high sensitivity (72.4%) and specificity (76.3%) in our study population. A validation study in a chronic disease out-patient clinic in Malawi also determined the optimal cut-of score to be nine, however the sensitivity was lower than in our study (64%) whilst the specificity was higher at 94% [30]. The performance of the PHQ-9 was similar to that from a range of other settings in sub-Saharan Africa [11, 12] and indicate that the PHQ-9 has reasonable sensitivity and specificity to be used as a screening tool for depression in this setting. Although the sensitivity and specificity values found in this study are not as high as found by Kroenke et al., (2001) this could be because of the use of the MINI as a gold standard; a recent meta-analysis showed that the sensitivity of the PHQ-9 was lower (0.77 versus 0.88) when using the MINI as the gold standard as compared to semi-structured interviews [32].

The construct validity of PHQ-9 was established by the factor analysis. All the items had factor loading in the range 0.54–0.82. Items related to low energy (0.82), feeling bad about self (0.72) and suicide (0.708) were most strongly related to the underlying construct meeting the diagnostic criteria for depression.

The PHQ-9 depression questionnaire appears to be a reliable and valid screening instrument for identifying depression in Botswana at a cut off score of nine or higher.

One of the strengths of this study is that it is the first to validate the PHQ-9 in Botswana. Another strength of our study includes the use of a clinical diagnostic gold standard to assess validity.

## Limitations

A limitation of this study is that the participants were drawn from only two primary health care facilities in the capital of Gaborone which may not be representative of the wider population. The use of convenience sampling presents a sampling error which limits generazibility of the findings.

## Conclusion

It is through primary health care services that mental health disorders can be observed and diagnosed. Thus, to improve health outcomes of patients in primary care, mental health disorders such as depression should be integrated into routine management. High burden of disease and negative impact on health outcomes, highlight this need for early diagnosis and management of depression. Despite being a middle-income country, poverty and high levels of income inequality persist and this has been found to predict depression in our population. Our study, similar to previous studies, demonstrates that the PHQ-9 is a reliable and valid instrument to screen for depression in primary care facilities in Botswana. The maximal specificity and sensitivity at a cut off score of 9 in our population compared to a cut off of 10 found in previous studies underscores the importance of validating the PHQ-9 in different socio-cultural populations. We recommend that, further studies should focus on integration of routine depression screening using the PHQ-9 in primary care.

## Data Availability

The datasets used and/or analysed during the current study are available from the corresponding author on reasonable request.

## Abbreviations

DSM: Diagnostic and Statistical Manual
MINI: Mini International Neuropsychiatric Interview
PHQ-9: Patient Health Questionnaire-9
WHOQoL-BREF: World Health Organisation Quality of Life Scale Brief Version
